# Insecticide-Treated Net Campaign and Malaria Transmission in Western Kenya: 2003–2015

**DOI:** 10.3389/fpubh.2016.00153

**Published:** 2016-08-15

**Authors:** Guofa Zhou, Ming-Chieh Lee, Andrew K. Githeko, Harrysone E. Atieli, Guiyun Yan

**Affiliations:** ^1^Program in Public Health, University of California Irvine, Irvine, CA, USA; ^2^Centre for Global Health Research, Kenya Medical Research Institute, Kisumu, Kenya

**Keywords:** insecticide-treated net, ownership, malaria parasite prevalence, vector density, cross-sectional survey, resurgence

## Abstract

Insecticide-treated nets (ITNs) are among the three major intervention measures that have reduced malaria transmission in the past decade. However, increased insecticide resistance in vectors, together with outdoor transmission, has limited the efficacy of the ITN scaling-up efforts. Observations on longitudinal changes in ITN coverage and its impact on malaria transmission allow policy makers to make informed adjustments to control strategies. We analyzed field surveys on ITN ownership, malaria parasite prevalence, and malaria vector population dynamics in seven sentinel sites in western Kenya from 2003 to 2015. We found that ITN ownership has increased from an average of 18% in 2003 to 85% in 2015. Malaria parasite prevalence in school children decreased by about 70% from 2003 to 2008 (the first mass distribution of free ITNs was in 2006) but has resurged by >50% since then. At the community level, use of ITNs reduced infections by 23% in 2008 and 43% in 2010, although the reduction was down to 25% in 2011. The indoor-resting density of the predominant vector, *Anopheles gambiae*, has been suppressed since 2007; however, *Anopheles funestus* populations have resurged and have increased 20-fold in some places since 2007. In conclusion, there is limited room for further increase in ITN coverage in western Kenya. The rebounding in malaria transmission highlights the urgent need of new or improved malaria control interventions so as to further reduce malaria transmission.

## Introduction

The scale-up of interventions, including insecticide-treated nets (ITNs), indoor residual sprays (IRS), and artemisinin-based combination therapy (ACT), has led to a significant reduction in malaria morbidity and mortality in the past decade (1). Long-lasting insecticidal nets (LLINs) have been a cornerstone of malaria control in the past several years, and millions are used each day across the globe (2). There is abundant literature about the success of the scale-up in suppressing malaria transmission, but there is also mounting evidence showing the limitations of these interventions (3–15). Factors limiting the effectiveness of ITNs include vector insecticide resistance due to selection pressure over time (16–19), outdoor biting and resting to avoid physical contact with ITNs (20–25), species shifting from mainly indoor-biting and resting species to more outdoor-resting species (11, 26), vectors biting animals (27), insecticide decay (28, 29), and varying durability of LLINs under different conditions of use (30).

Kenya is one of the south Saharan African countries where malaria is most prevalent (1). Western Kenya currently has the highest malaria transmission intensity in the country (1, 31). Similar to the rest of the world, the major ITN, IRS, and ACT campaign in Kenya began in 2006 (11). ITN coverage was low in Kenya before 2006, although retail and subsidized ITN campaigns were initiated in early 2000 (11). In 2006, the Ministry of Health began mass distribution of free ITNs to children under 5 years and pregnant women (11, 30). In 2011, the policy changed to cover the entire at-risk population regardless of age and gender (30). In 2014, the third round of mass LLIN distribution was launched to boost ITN coverage and replace old ITNs (30). These mass campaigns significantly increased overall ITN coverage (14, 30). However, evidence shows a mixed picture of the success of the current scaled-up antimalarial campaign. While some earlier studies reported a continuous decline in malaria transmission in selected sites (32–35), more recent studies show that malaria transmission has remained unchanged or has even resurged in western Kenya (11, 14). Possible risk factors limiting the effectiveness of ITNs have been explored, including vector insecticide resistance, outdoor transmission, vector species shifting, vectors biting animals, insecticide decay, and the varying integrity of LLINs under different conditions of use (14, 18, 25, 27, 28). However, no study has looked into the effectiveness of ITNs over time to compare the impact of ITNs on malaria transmission at different periods – e.g., before or immediately after the first mass ITN campaign in 2006 compared with now, about 10 years later. This knowledge would be valuable to malaria control programs as they plan for procurement and replacement.

The aim of this study is to explore the changes in ITN coverage since 2003, 3 years before the scaled-up antimalarial campaign began, and compares the effectiveness of ITNs on vector indoor-resting density and parasite prevalence over time (i.e., before, immediately after, and about 10 years after the first mass ITN campaign). We aim to determine whether adherence to, and public health benefits of, insecticide-treated bednets can be sustained over time, i.e., maintains malaria vector population and parasite prevalence at low levels.

## Materials and Methods

### Study Area

**Figure 1:**
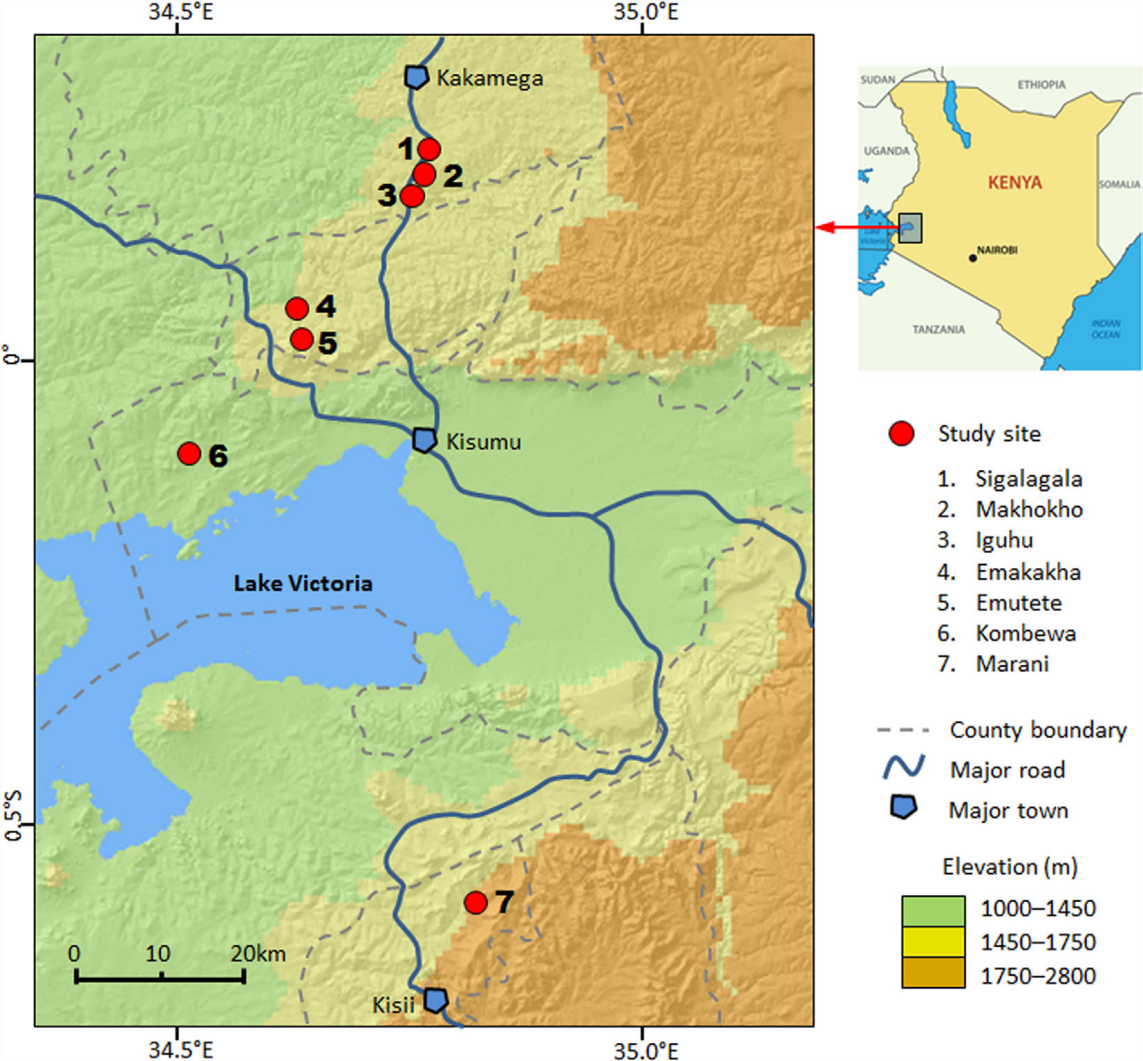
**Map of sampling sites in western Kenya**.

This study was conducted in seven sites in four counties in western Kenya: Kakamega County, Vihiga County, Kisumu County, and Kisii County (Figure 1). The study area includes both high- and low-transmission sites and both highlands and lowlands (Table S1 in Supplementary Material) (18, 25, 28, 30). The details of climate, geography, and malaria transmission in the area have been described elsewhere (11, 25, 30). Briefly, all study sites are located in hilly areas, with altitudes ranging from 1180 m above sea level (a.s.l.) in Kombewa to 1770 m a.s.l. in Marani (Figure 1). Each site has distinct topology, but all seven sites are characterized by valleys surrounded by hills of different slopes. Climate in the study area consists mainly of two seasons of rainfall: a long season between March and May (the peak malaria transmission season) and a short one between October and November.

### ITN Ownership and Usage Survey

Insecticide-treated net ownership was surveyed in three villages: Iguhu, Kombewa, and Marani. The detailed field surveys have been described in previous studies (30). Briefly, ITN ownership and usage surveys were conducted along with monthly entomological surveillance. This arrangement saved time and money and avoided logistical issues, such as transportation and manpower; this method has been validated by cross-sectional surveys (30). Houses at each site were randomly selected for participation. Owners of the selected houses were requested to sign a freely administered informed consent form covering participation in the study, questionnaire surveys of ITN ownership and monitoring of ITN conditions, and demographic information. ITN ownership was surveyed in 2003, 2006, and every year from 2008 to 2015.

### Malaria Vector Surveys

Malaria vectors were sampled in Iguhu, Kombewa, and Marani in 2003, 2008, 2011, and 2015 (Tables S1 and S2 in Supplementary Material). Pyrethrum spray collections were conducted monthly in 30 randomly selected sentinel houses in each village for 12 months each year. The houses were chosen to cover at least three elementary school areas. The selection of houses to be sampled was re-randomized each month, but the sampled houses were all located in the same general area from 2003 to 2015. The presence or absence of ITNs in each house was also confirmed and recorded during mosquito sampling. Mosquitoes collected were morphologically identified as *Anopheles gambiae* s.l. or *Anopheles funestus*.

### Cross-Sectional Asymptomatic Parasite Screening

Asymptomatic malaria parasite infection surveys were conducted in different communities in 2006, 2010, and 2011 (Tables S1 and S2 in Supplementary Material). Surveys were done once a year during the high-transmission months of May and June. The surveys included all ages. To maximize the representativeness of the samples, the sampling area was stratified to cover three elementary schools in each study site, and participants were selected randomly from each school catchment area. Upon signing the informed consent (assent for minors under age 18) forms, participants completed a short questionnaire survey that included demographic and ITN ownership/usage information. Blood samples were collected by the standard finger-prick method (11), and thin and thick blood smears were prepared for laboratory examination. Parasite species and gametocytes were identified microscopically. Malaria parasite counts were read against 200 white blood cells, and density was expressed as parasites per milliliter assuming a count of 8,000 white blood cells per microliter. All slides were examined by two experienced laboratory technicians at Kenya Medical Research Institute (KEMRI) to identify the parasite species. For quality control purposes, a third technician randomly selected 5% of the slides for re-examination.

### Parasite Screening in School Children

Parasitological surveys were conducted with randomly selected school children aged 6–13 years in Iguhu, Kombewa, and Marani (Tables S1 and S2 in Supplementary Material) (11). Data used in this study included February, April, and June surveys from 2003, 2008, 2011, and 2015. During each sampling occasion at each site, at least 100 volunteer school children from 5to 6 primary schools were sampled to determine parasite prevalence. The catchment area at all sites remained unchanged over time. During high-transmission season (June of each year), we increased the sample size to 300 volunteers to increase the statistical power. Blood sample collection, slide preparation, parasite identification, and parasitemia counts were conducted as described in the previous section. This survey was purely for parasite screening; no questionnaire was administered.

### Data Analysis

Household ITN ownership was calculated as the percentage of households with at least one ITN during the survey. Parasite prevalence was calculated as the percentage of positive cases over total slides examined, based on pooling all surveys in each year at each site. Indoor-resting malaria vector density was calculated as females per house per night, pooled by month at each site. Percentage reduction in indoor-resting vector density between households with ITN and without ITN was calculated as

$$\frac{\left(\text{mean density in households with ITNs})-(\text{mean density in households without ITNs}\right)}{\left(\text{mean density in households with ITNs}\right)}\times 100.$$

Differences in vector densities between different years and between ITN owners and non-owners were compared using a *t*-test or ANOVA. Differences in parasite prevalence between different years were compared using a χ^2^ test.

### Scientific and Ethical Clearance

Scientific and ethical clearance was given by the institutional review boards (IRBs) of KEMRI, Kenya, and the University of California at Irvine, USA. Volunteers were enrolled from primary schools in the study sites through school administrators with the permission of the division office of the Ministry of Health. Written assent for children (<18 years of age) was obtained by the participants and their parents or guardians. Written consent and assent for households was obtained from the head of the household. Inclusion criteria included providing informed consent and having no reported chronic or acute illness except malaria. Exclusion criteria included unwillingness to participate in the study. According to the standard malaria treatment guidelines of the Ministry of Health of Kenya, asymptomatic infections were not treated with antimalarials. Symptomatic volunteers were referred to the local government hospital or clinic for diagnosis and treatment free of charge.

## Results

**Figure 2:**
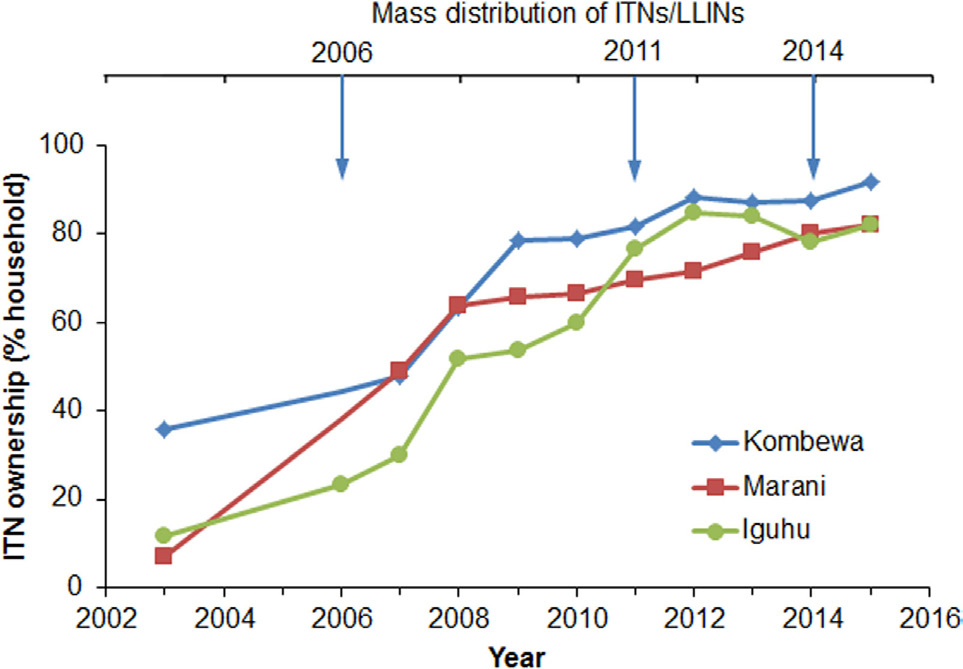
**Dynamics of ITN ownerships from 2003 to 2015 at three study sites in western Kenya**.

Household ITN ownership increased from an average of 18.3% in 2003 to 85.3% in 2015 (Figure 2). The sharpest increase in ITN ownership occurred from 2006 to 2011, and the positive trend began to slow in 2012 (Figure 2). Although there was a mass ITN distribution in the study area in 2014, ITN ownership only increased by about 3% from 2013 to 2015. In fact, ITN ownership has decreased by about 4% in Iguhu from 2012 to 2015 (Figure 2). Presumably the 2014 ITN campaign mainly replaced old ITNs as opposed to introducing ITNs into homes that had not had them before.

**Figure 3:**
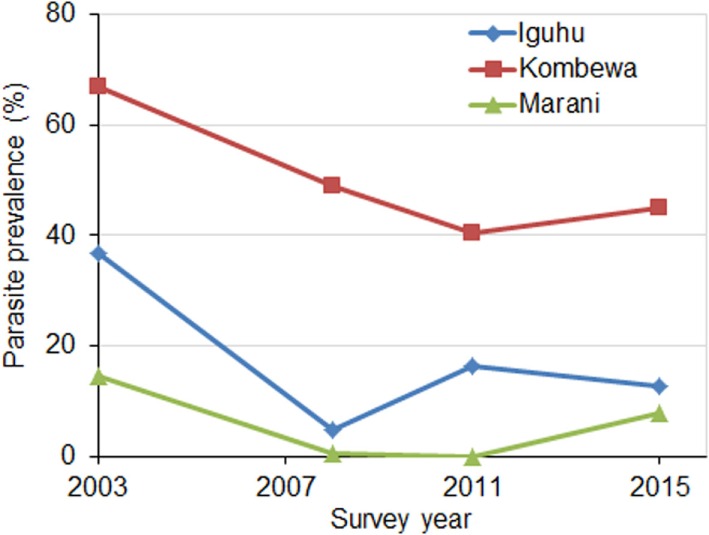
**Parasite in school children from 2003 to 2015 at three study sites in western Kenya**.

Parasite prevalence in school children decreased sharply at all sites from 2003 to 2008 (χ^2^ tests, *P* < 0.01 at all sites) (Figure 3). The average reduction in parasite prevalence in this period was 70.5%. Parasite prevalence dropped continuously from 2008 to 2011in two sites, whereas it increased twofold in one site (Figure 3). Compared to 2008, parasite prevalence in 2015 had increased by an average of 57.5%.

Compare to non-users, use of ITNs reduced community-wide parasite prevalence by 23.6% in 2006. The reduction in parasite prevalence by the use of ITNs compare to non-users increased to 43.3% in 2010 but decreased to 25.6% in 2011 (Table 1).

**Table 1:** **ITN coverage and differences in parasite prevalence between ITN users and non-users**.

Indicator		Survey year		
		2006	2010	2011
ITN usage		23.30 ± 1.08	43.77 ± 8.44	85.59 ± 14.01
Parasite prevalence	Overall (%)	15.12 ± 9.63	12.88 ± 6.18	11.73 ± 9.95
	With ITN (%)	11.51 ± 5.65	9.07 ± 4.83	11.22 ± 10.56
	No ITN (%)	16.17 ± 10.74	16.28 ± 7.47	13.66 ± 4.59
	Reduction (%)	23.60 ± 13.44	43.32 ± 13.22	25.63 ± 47.71

**Figure 4:**
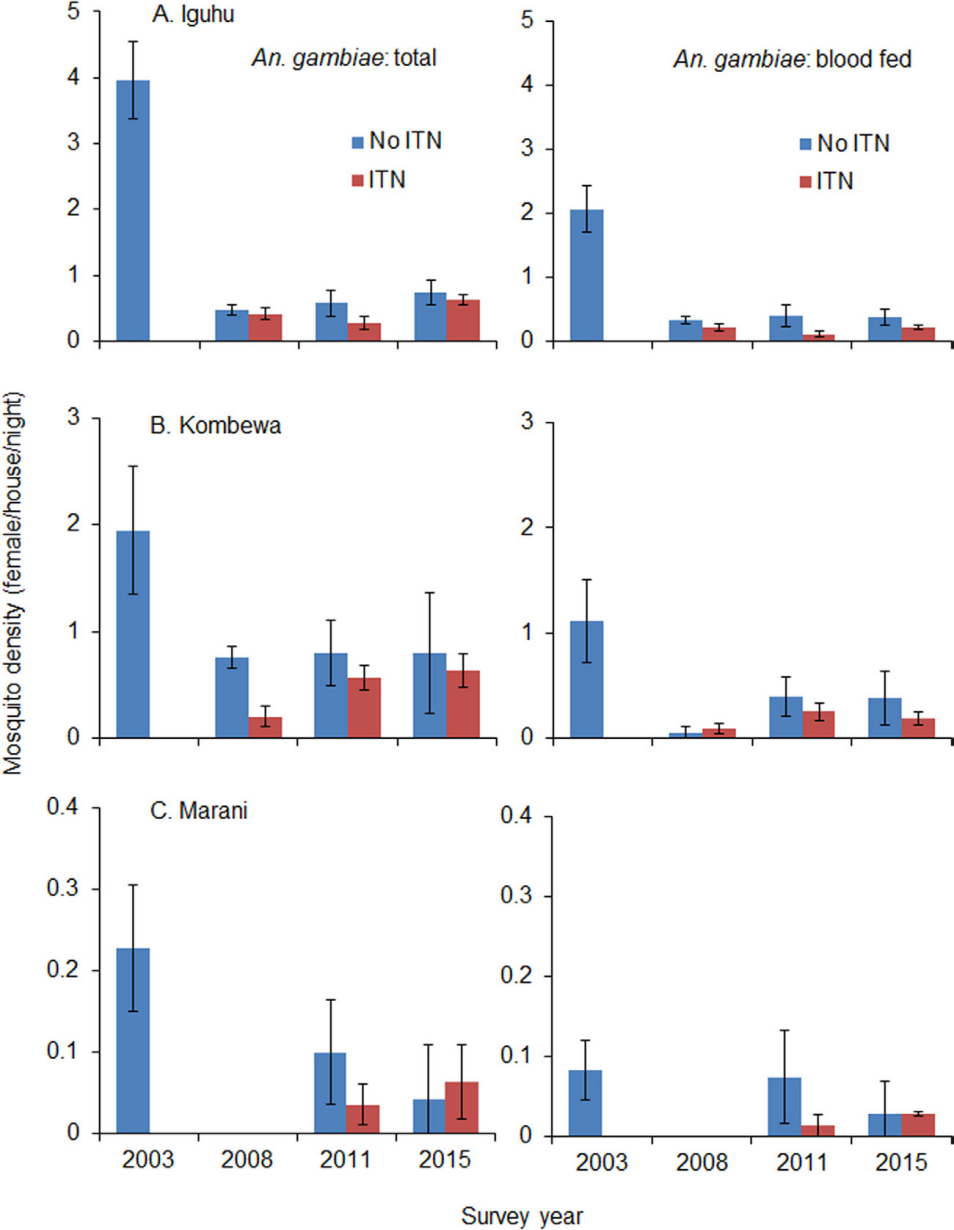
**Impact of ITN usage on indoor-resting malaria vector densities from 2003 to 2015 at three study sites in western Kenya**. **(A)** Iguhu, **(B)** Kombewa, **(C)** Marani.

**Figure 5:**
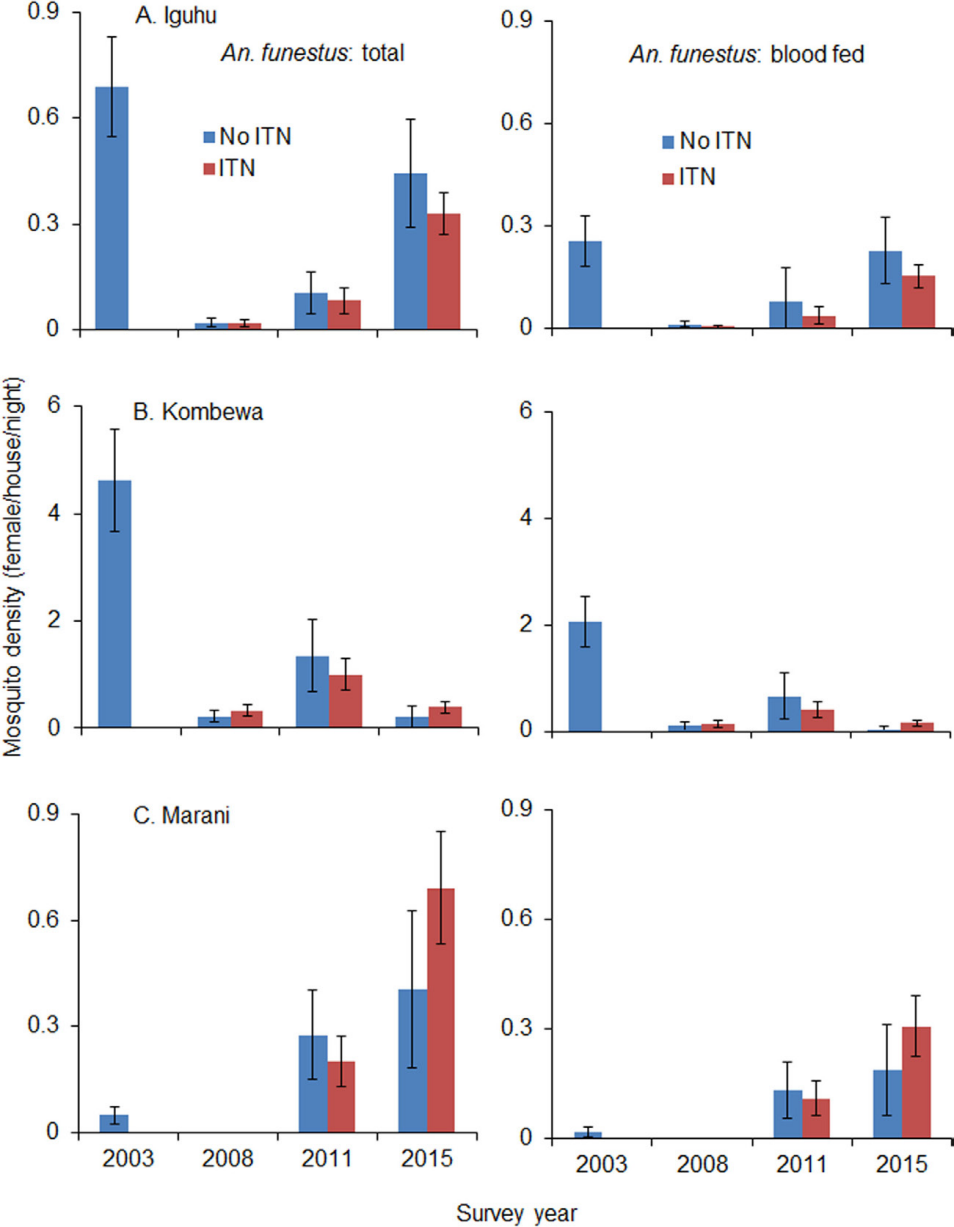
**Impact of ITNs on *A. funestus* densities over time at (A) Iguhu, (B) Kombewa and (C) Marani**.

Total vector density was 82.9% lower on average in 2008 compared to 2003 (ranging from 61.0 to 100% lower), while blood-fed vector density decreased by 93.4% (ranging from 84.2 to 100%). Vector density was significantly lower in 2008 compared to 2003 regardless of species and study site (Figure 4). The pyrethrum spray collection method did not find any indoor-resting malaria vectors in Marani in 2008. Since 2008, the density of indoor-resting *A. gambiae* s.l. has remained unchanged in Kombewa and Iguhu but has resurged in Marani, although it is still significantly lower than in 2003 (Figure 4). By contrast, the density of *A. funestus* has rebounded significantly at all sites since 2011 (Figure 5), and in Marani *A. funestus* density was 20-fold higher in 2015 (1.02 females/house/night) compared to 2003 (0.05 females/house/night) (unequal variance *t*-test, *t* = 6.10, d.f. = 7, *P* < 0.001).

Use of ITNs reduced indoor-resting *A. gambiae* s.l. density by an average of 36.8% in 2008 compare to non-users, but the reduction decreased to 9.9% in 2015 (Table 2). Use of ITNs did not reduce indoor-resting *A. funestus* density regardless of ITN coverages, i.e., in both 2008 when ITN coverage was low and 2015 when ITN coverage was high. Reduction in blood-fed density due to ITNs was insignificant regardless of vector species and ITN coverages (Table 2).

**Table 2:** **Percentage reduction in indoor-resting vector density between households with ITN and without ITN**.

Year	*A. gambiae*		*A. funestus*	

	Total	Blood-fed	Total	Blood-fed
2008	36.8	−48.9	−25.3	−22.3
2011	13.6	16.7	11.6	16.9
2015	9.9	24.8	−22.7	−46.9

## Discussion

Insecticide-treated nets have proven to be one of the most effective tools for malaria control (1, 2, 36–39). In the 1990s, ITNs sustainably reduced malaria transmission, especially malaria-specific mortality, when they were first introduced into communities (32, 36). Over the past decade, ITNs, together with other interventions, have saved millions of lives and effectively reduced malaria transmission all over the world (1, 2). However, recent studies show a resurgence in malaria transmission despite high ITN coverage (7, 14). While there has been limited epidemiologic data demonstrating that pyrethroid resistance reduces the effectiveness of ITNs, several studies conducted in pyrethroid-resistant areas have demonstrated the continued ability of ITNs to protect against malaria transmission when properly deployed (19, 40–42). Conclusions from different studies can be contradictory; some studies conclude that ITNs did not provide significant personal protection (43), while studies from the same area suggest that ITNs significantly reduced the incidence of malaria infection (44). This study shows that ITNs still have a significant community-wide effect on reducing the overall parasite prevalence, despite the resurgence of indoor-resting vectors.

Although recent studies and meta-analysis of entomologic outcomes have shown that ITNs continued to reduce blood feeding and increase mosquito mortality even in areas with the highest levels of resistance (45), this study did not find significant reduction in indoor-resting vector densities in households with ITNs compared to households without ITNs. The sharp decrease in indoor-resting vector density in 2008 is likely due to a community-wide effect when insecticide resistance was low. The stability of indoor-resting *A. gambiae* s.l. may be due to three factors: ITNs remaining effective against *A. gambiae* s.s., vector species shifting toward *Anopheles arabiensis*, and vectors feeding and resting outdoors (11, 18, 25–27). *A. gambiae* s.l. has definitely developed resistance to pyrethroid insecticides in western Kenya, but insecticides still kill over 70% of mosquitoes when tested against the WHO tube bioassay (18). On the other hand, *A. arabiensis* has been increasing in the study area relative to *A. gambiae* s.s. (11, 25). For example, in Kombewa, *A. arabiensis* accounted for <5% of *A. gambiae* s.l. in 2003 but had increased to 74% in 2014 (11, 25). *A. arabiensis* rests outdoors more often and feeds on both humans and animals (27). Outdoor transmission has also been observed in the study area (14, 25, 27). The increase in indoor-resting *A. funestus* density is likely due to insecticide resistance, insecticide decay, and a lack of physical durability in LLINs (28, 30, 46–48).

## Conclusion

Insecticide-treated nets still have a significant community-wide effect on reducing overall parasite prevalence. The use of ITNs is still effective against *A. gambiae* s.s. but it faces the challenges of species shift and increasing insecticide resistance and outdoor transmission. The resurgence of indoor-resting *A. funestus* indicates that *A. funestus* may be more resistant to ITN use and potentially become the most important malaria vector in western Kenya. The shift in pattern of mosquito species from *A. gambiae* to *A. funestus*, which can survive on both human and animal blood, therefore, may pose a problem in future, this situation must be closely monitored. There is also a need to continuously evaluate the use of ITN and insecticide resistance. While the causes may be complex, all evidence points toward the reduction in malaria transmission reaching a plateau in the study area. This calls for the development and adoption of viable alternative methods of malaria vector control that can reduce the reliance on pyrethroids.

## Author Contributions

Designed the study: GZ and GY; performed statistical analysis and data preparation: GZ; contributed to data collection: GZ, M-CL, HA, and AG; wrote the paper: GZ and GY; and contributed to the final version and interpretation of results: GZ, AG, and GY.

## Conflict of Interest Statement

The authors declare that the research was conducted in the absence of any commercial or financial relationships that could be construed as a potential conflict of interest.
