# Supplementary material for: Insecticide-Treated Net Campaign and Malaria Transmission in Western Kenya: 2003–2015
Source: Front Public Health. 2016 Aug 15;4:153. doi: 10.3389/fpubh.2016.00153 (PMC4983699; doi:10.3389/fpubh.2016.00153)
Supplement: Supplementary file 1 [file Table_1.DOCX]

Additional file 1

Table S1. Locations of study sites.

| County | Study site ^†^ | Location (longitude/latitude) | Altitude (m) |
| --- | --- | --- | --- |
| Kakamega | 1. Sigalgala | 34.76°E, 0.20°N | 1550-1600 |
|  | 2. Makhokho | 34.75°E, 0.18°N | 1480-1530 |
|  | 3. Iguhu | 34.74°E, 0.16°N | 1430-1580 |
| Vihiga | 4. Emakakha | 34.63°E, 0.08°N | 1450-1530 |
|  | 5. Emutete | 34.65°E, 0.03°N | 1540-1620 |
| Kisumu | 6. Kombewa | 34.52°E, 0.11°S | 1210-1320 |
| Kisii | 7. Marani | 34.80°E, 0.58°S | 1540-1760 |

^†^ Refer to Figure 1 for site numbers and site names

Meteorological station data, Kisumu station for Rae, Miwani and Kombewa; Kakamega station for Iguhu and Emakaha; and Kisii station for Marani.
