# Supplementary material for: Insecticide-Treated Net Campaign and Malaria Transmission in Western Kenya: 2003–2015
Source: Front Public Health. 2016 Aug 15;4:153. doi: 10.3389/fpubh.2016.00153 (PMC4983699; doi:10.3389/fpubh.2016.00153)
Supplement: Supplementary file 2 [file Table_2.DOCX]

Additional file

Table S2. Sample size at each sampling site by indicators and years

| County | Site | Parasite prevalence sampling (individuals) | | | | | | |  | Mosquito sampling (Households) | | | |
| --- | --- | --- | --- | --- | --- | --- | --- | --- | --- | --- | --- | --- | --- |
|  |  | Community (all age) | | | School children (5-14 yrs) | | | |  |  |  |  |  |
|  |  | 2006 | 2010 | 2011 | 2003 | 2008 | 2011 | 2015 |  | 2003 | 2008 | 2011 | 2015 |
| Kakamega | 1. Sigalagala | 485 | 273 |  |  |  |  |  |  |  |  |  |  |
|  | 1. Makhokho | 477 | 369 |  |  |  |  |  |  |  |  |  |  |
|  | 1. Iguhu | 503 | 389 | 1355 | 1805 | 550 | 636 | 413 |  | 552 | 330 | 388 | 388 |
| Vihiga | 1. Emakakha |  | 545 | 1608 |  |  |  |  |  |  |  |  |  |
|  | 1. Emutete |  | 645 | 1642 |  |  |  |  |  |  |  |  |  |
| Kisumu | 1. Kombewa |  |  |  | 500 | 843 | 648 | 632 |  | 208 | 185 | 388 | 424 |
| Kisii | 1. Marani |  |  |  | 605 | 525 | 551 | 487 |  | 333 | 200 | 393 | 384 |
